# Radiofrequency ablation of a reentrant atrial tachycardia at the superior left atrium via the pulmonary artery

**DOI:** 10.1016/j.hrcr.2025.10.005

**Published:** 2025-10-10

**Authors:** Leon Dinshaw, Andreas Koltzau, Dominik Linz, Kevin Vernooy, Marten Grau, Youssef Benslama

**Affiliations:** 1 Department of Cardiology, Sana Hanse Klinikum, Wismar, Germany; 2 Department of Cardiology, Maastricht University Medical Centre and Cardiovascular Research Institute Maastricht, Maastricht, The Netherlands; 3 Abbott Medical GmbH, Eschborn, Germany

**Keywords:** Atrial tachycardia, Epicardial ablation, Roof-dependent atrial tachycardia, Pulmonary artery ablation, Reentrant atrial tachycardia mapping

**Key Teaching Points**

• Despite high-resolution mapping catheters, the identification of electrical signals of the critical isthmus is sometimes not possible. In this case, classic electrophysiological mapping strategies such as entrainment are necessary for understanding complex atrial tachycardias.
• Epicardial pathways at the left atrial roof possibly play a critical role in localized reentry tachycardias with difficult endocardial ablation. Understanding the anatomic proximity of the pulmonary arterial system may help to effectively treat these tachycardias with radiofrequency energy.
• Further studies are justified to verify the effectiveness and safety of radiofrequency ablation via the pulmonary artery.

## Introduction

Reentrant atrial tachycardias (ATs) are a frequent and challenging arrhythmia, particularly in patients with atrial cardiomyopathy and/or previous ablation of atrial fibrillation (AF).^1^ Reentrant ATs generally are categorized into localized reentrant AT (LRAT) and macroreentrant AT (MRAT).^2^^,^^3^ Their occurrence is often facilitated by anatomic and/or atrial substrates, such as atrial scarring. High-density mapping techniques often in conjunction with classic electrophysiological diagnostics, such as entrainment mapping, have advanced the understanding of the reentrant cycle and its critical isthmus, thereby aiding successful ablation with a favorable long-term outcome.^4^^,^^5^ Roof-dependent MRAT and LRAT in the superior left atrium (LA) are potentially challenging owing to the anatomic intricacies of the superior LA and the involvement of epicardial fibers.^6^^,^^7^

We report the case of an 81-year-old woman presenting with symptomatic AT after previous catheter ablation of AF. The arrhythmia was refractory to medical therapy, and therefore, electrophysiological study with catheter ablation was indicated.

## Case report

An 81-year-old woman with previous catheter ablation of AF with a pulmonary vein (PV) isolation and a linear ablation at the LA roof using radiofrequency (RF) energy in May 2024 presented with symptomatic AT. During the index procedure, the anterior line had demonstrated bidirectional block, owing to extensive scarring. The patient had a normal left ventricular ejection fraction, mild LA dilatation (55 mL), a history of hypertension, and a previous stroke.

**Figure 1:**
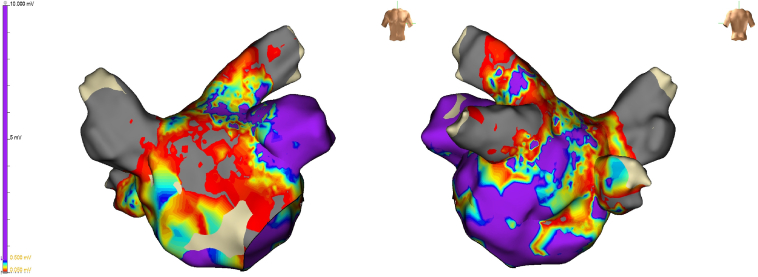
Electroanatomic high-density omnipolar voltage map showing large low-voltage areas at the anterior and superior left atrium during mapping of the initial atrial tachycardia (AT1). AT = atrial tachycardia.

**Figure 2:**
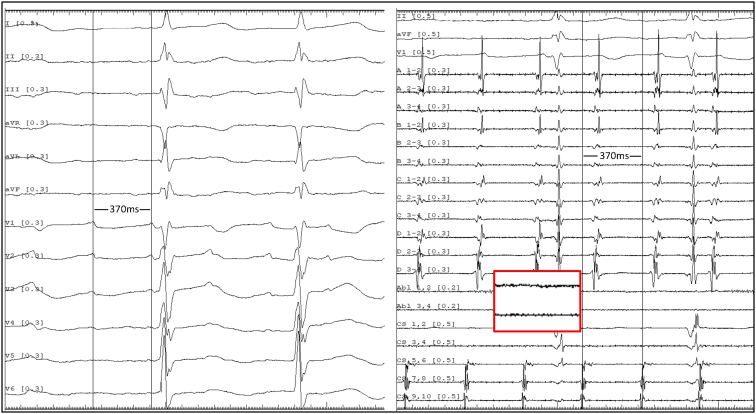
Twelve-lead ECG of the second atrial tachycardia (AT2) (left) and intracardiac electrograms from mapping and ablation catheters (right). The tachycardia cycle length was 370 ms with concentric activation in the CS. The HD Grid catheter was positioned in the LAA, whereas the ablation catheter was located in the pulmonary artery at the site where RF delivery immediately terminated AT2. No discernible electrograms were recorded at this location despite high amplification (magnified in the *red box*). AT = atrial tachycardia; CS = coronary sinus; ECG = electrocardiogram; LAA = left atrial appendage; RF = radiofrequency.

**Figure 3:**
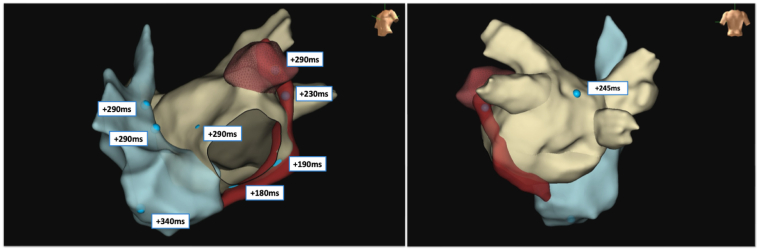
Entrainment mapping during the second atrial tachycardia (AT2). PPI–TCL values are shown for multiple atrial pacing sites, including the lateral and inferolateral LA, anterior wall, proximal/middle/distal CS, cavotricuspid isthmus, and right atrial septum. All sites demonstrated long PPI–TCL values, indicating the absence of concealed entrainment and exclusion from the tachycardia circuit. Pacing from the superior endocardial LA failed to achieve myocardial capture, resulting in ineffective entrainment. AT = atrial tachycardia; CS = coronary sinus; LA = left atrium; PPI–TCL = postpacing interval minus tachycardia cycle length.

**Figure 4:**
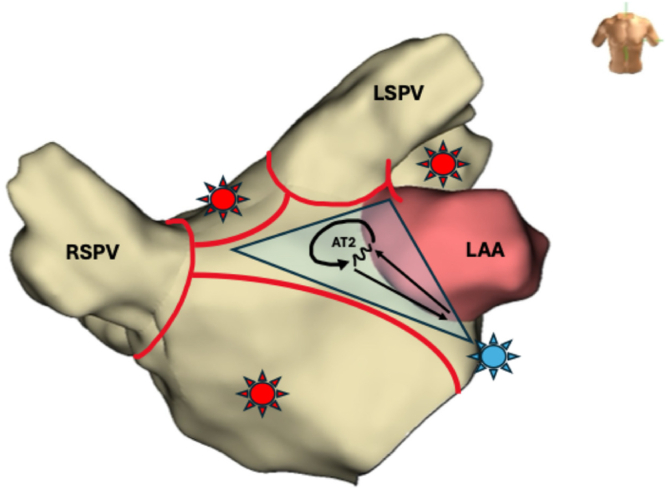
Anterosuperior view of the LA showing the presumed localized reentry circuit of the second atrial tachycardia (AT2) (*black arrow*) at the superior LA. Blocked lines identified by entrainment mapping are shown in *red*, forming a triangular region (*transparent blue*) bounded by the anterior line, the posterior roof line, and the ridge, along with pulmonary vein isolation lines. Pacing sites are marked with asterisks: the shortest PPI–TCL (∼180 ms) was recorded at the inferolateral LA (*blue asterisk*), whereas very long PPI–TCL values (∼230–290 ms) were observed at the posterior and anteroseptal LA and in the distal coronary sinus (*red asterisks*). These findings suggest that the critical isthmus was confined to the superior LA, likely beneath the pulmonary artery. AT = atrial tachycardia; LA = left atrium; LAA = left atrial appendage; LSPV = left superior pulmonary veins; PPI = postpacing interval; RSPV = right superior pulmonary vein; TCL = tachycardia cycle length.

**Figure 5:**
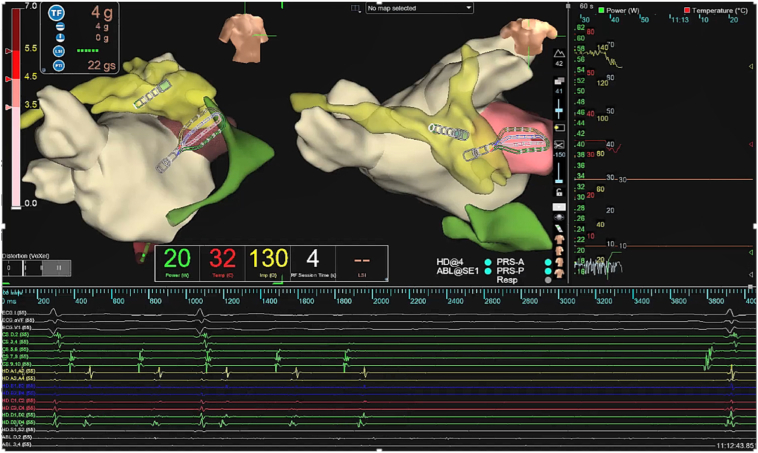
Immediate termination of the second atrial tachycardia (AT2) during epicardial-targeted ablation from the pulmonary artery, confirming a localized epicardial circuit of the tachycardia at the left atrial roof.

At the time of the current electrophysiological study, surface electrocardiogram showed a regular AT. Vascular access was obtained via the right femoral vein. Two Swartz SL1 transseptal sheaths (Abbott, Chicago, IL) and a third sheath (Radifocus Introducer II, Terumo, Tokyo, Japan) were inserted. A decapolar diagnostic catheter was positioned in the distal coronary sinus (CS) and demonstrated eccentric activation of the LA with a cycle length (CL) of 310 ms (first AT [AT1]). After transseptal access, unfractionated heparin (100 IE/kg) was given, and during LA dwell time, an activated clotting time of 300–350 seconds was targeted. Three-dimensional electroanatomic modeling using the EnSite X system (Abbott, St. Paul, MN) was performed. High-density mapping with the Advisor HD Grid Mapping Catheter (Abbott, St. Paul, MN) revealed a mildly dilated LA with extensive low-voltage areas at the anterior and superior LA walls (Figure 1). Activation mapping confirmed a macroreentrant circuit around the left PVs, with a critical isthmus at the superior LA near the ridge, adjacent to the previous roof line, and an endocardially blocked septopulmonary bundle (Supplemental Video 1). RF ablation at this site eventually transformed AT1 (CL 310 ms) into a slower second AT (AT2) (CL 370 ms), suggesting circuit modification or a new reentry. During this process, reconnection of the left PVs was eliminated, and the right inferior PV was isolated by ablation of a posterior gap. The 12-lead electrocardiogram is presented in Figure 2. However, activation mapping of the second AT2 did not show a conclusive reentrant circuit given that extensive endocardial low-voltage areas at the superior and anterior LA did not reveal appropriate signal identification despite the use of a multipolar mapping catheter designed to reduce bipolar blindness. Ablation with an irrigated RF catheter (TactiCath SE, Abbott, St. Paul, MN) at the superior LA repeatedly resulted in termination of the AT2 into sinus rhythm, but reinduction was easily possible. High-power, short-duration (50 W for up to 13 seconds) and standard RF (30 W for 25–45 seconds) settings were used. Extensive entrainment mapping maneuvers were performed, and values of postpacing intervals (PPIs) subtracted by the tachycardia CL (TCL) at multiple locations in the left and right atrium were compared (Figure 3). Although no effective entrainment could be obtained at the superior LA because pacing failed to capture, the overall findings were highly informative. Long PPI–TCL values at the anterior septum, posterior LA, and distal CS excluded perimitral/septal, posterior roof–dependent, and Marshall ligament tachycardias, respectively. These results indicated conduction block across the anterior line, the posterior roof line, and the ridge, leaving only a small triangular region at the superior LA as the potential corridor for conduction (Figure 4). The apex of this triangle pointed toward the anterolateral mitral annulus near the LA appendage, where the shortest PPI–TCL was observed at the middle CS and inferolateral LA, although still relatively long (∼180 ms). This suggested that the true isthmus lies at some distance, most likely in the superior LA, given that all other pacing sites showed even longer PPI–TCL values. Despite the absence of endocardial electrograms in the superior wall, the consistent termination into sinus rhythm with endocardial ablation indicated that this confined region was the likely site of a localized reentry. The lack of effect from further endocardial lesions strongly suggested that the critical isthmus was protected and epicardial in nature. Importantly, the suspected reentry was confined to a relatively small area, anatomically situated immediately beneath the pulmonary artery. Access to the pulmonary artery was obtained to target the presumed epicardial site over the LA roof. No local electrograms were detected (Figure 2), and high-output pacing failed to capture myocardium. Nevertheless, entrainment findings and the reproducible termination of AT2 with endocardial ablation at the adjacent superior LA strongly suggested that this area contained a critical component of the circuit. These observations convinced us that empiric ablation from within the pulmonary artery at this location was reasonable. Irrigated RF ablation (20 W, 17 mL/min) from the pulmonary artery led to immediate termination of AT2 and restoration of sinus rhythm (Figure 5; Supplemental Video 2). RF energy was titrated to 25 W and continued for 35 seconds, followed by minor additional ablation in the region. Empirical cavotricuspid isthmus ablation was also performed. Final assessment confirmed durable PV isolation, intact roof and anterior lines, and no inducible arrhythmia after burst pacing (minimum CL 240 ms, up to 20 beats). The procedure was completed without complications. Oral anticoagulation was continued per protocol in a patient with AF and AT.

## Discussion

ATs remain a significant clinical challenge, particularly when multiple circuits develop in atria with extensive intrinsic low-voltage areas^8^^,^^9^ or in patients with iatrogenic low-voltage areas after previous AF ablation. In this case, sequential ATs emerged during ablation at the superior LA, reflecting the trend toward complex reentrant circuits in this region. The AT1 was characterized as an MRAT with its critical endocardial isthmus at the superior ridge successfully terminated by RF ablation. However, the AT2 resisted endocardial RF ablation. Absence of local electrograms, failed myocardial capture upon high-output pacing (20 mA/0.5 ms), and ineffective endocardial RF ablation pointed toward an arrhythmia mechanism involving an epicardial substrate. Entrainment mapping revealed blocked lines at the anterior LA, the posterior roof line, and the ridge, suggesting an LRAT at the superior LA involving an epicardial circuit. Epicardial fibers such as the septopulmonary bundle, Bachmann’s bundle, or remnants of the vein of Marshall have been implicated in similar roof-dependent ATs, especially when standard endocardial lesions fail to achieve arrhythmia termination.^10^, ^11^, ^12^ Previous cases have been reported in which ablation via the pulmonary artery effectively treated AT after multiple unsuccessful attempts from the endocardial LA.^13^^,^^14^ In our case, RF ablation from the pulmonary trunk (20–25 W, 17 mL/min irrigated flow) led to immediate AT termination. Despite the absence of electrograms and ineffective myocardial capture, this bailout approach proved effective. This strategy highlights the value of targeted epicardial ablation of the LA roof in selected cases.

**Figure 6:**
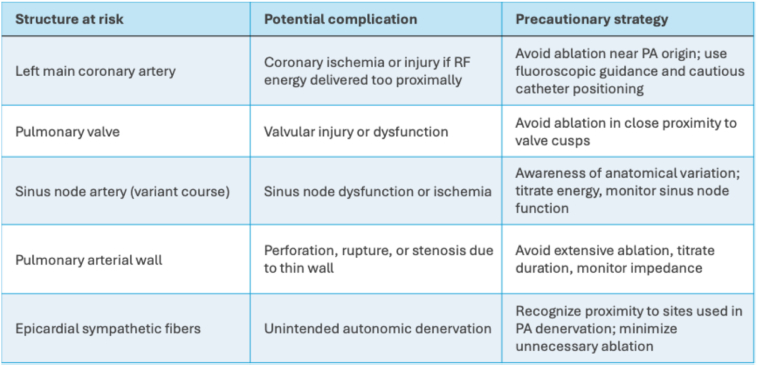
Key anatomic risks during ablation from the pulmonary artery. Vulnerable structures include the left main coronary artery, pulmonary valve, sinus node artery, pulmonary arterial wall, and epicardial sympathetic fibers. Awareness of these sites and titrated RF delivery are essential for procedural safety. RF = radiofrequency.

The thin wall of the pulmonary artery predisposes it to perforation, rupture, or stenosis. Although we did not assess structural changes post-ablation, RF ablation in the PA for sympathetic denervation in pulmonary hypertension has been reported with acceptable safety profiles.^15^ The clinical significance of unintended sympathetic fiber injury during atrial arrhythmia ablation from the PA remains uncertain, and long-term outcome monitoring is warranted. Other potential risks include the proximity of the left main coronary artery and pulmonary valve cusps in the proximal PA, as well as variant courses of the sinus node artery with a potential risk of sinus node dysfunction. Ablation of ventricular arrhythmias from the supravalvular apparatus and pulmonary cusps has generally been considered safe, although proximity to the coronary arteries requires caution.^16^^,^^17^ A single case report has described ablation near a sinus node artery with a variant origin from the left coronary system, underscoring the need for careful monitoring of sinus node function, particularly when larger lesion sets are applied, such as for completion of an anterior line.^18^^,^^19^ Direct epicardial ablation of atrial arrhythmias has also been described, but may carry a higher risk of adverse events.^20^^,^^21^ Key anatomic risks, potential complications, and precautionary strategies are presented in Figure 6.

In addition to RF ablation, newer technologies such as focal pulsed field ablation may offer alternative solutions in challenging arrhythmia mechanisms and cardiac anatomies^22^; however, studies are required to assess the expected myocardial lesion depth of focal pulsed field ablation from within the pulmonary artery.^23^^,^^24^ Transition of multiple tissue levels (pulmonary arteria wall, fat, myocardium) may reduce myocardial lesion depth after focal pulsed field ablation, as also demonstrated for ablation from within the great cardiac vein.^25^

This case underscores the importance of detailed anatomic knowledge in managing roof-dependent ATs unresponsive to conventional strategies. The anatomic proximity of the pulmonary artery to the superior LA may allow for effective epicardial targeting when standard endocardial approaches fail.

## Conclusion

In cases of ATs at the superior LA refractory to endocardial ablation, epicardial pathways may play a critical role. This case illustrates that ablation via the pulmonary artery can serve as an effective adjunctive strategy when conventional approaches fail. A comprehensive understanding of atrial anatomy, precise mapping, and adaptive interventional techniques is essential for successful management in such complex scenarios.

## Declaration of generative AI and AI-assisted technologies in the writing process

During the preparation of this work, the authors used ChatGPT-4o for language refinement. After using this tool/service, the authors reviewed and edited the content as needed and take full responsibility for the content of the publication.

## Disclosures

The authors have no conflicts of interest to disclose. The other authors have no conflicts of interest to disclose.
