# Supplementary material for: Radiofrequency ablation of a reentrant atrial tachycardia at the superior left atrium via the pulmonary artery
Source: HeartRhythm Case Rep. 2025 Oct 10;11(12):1373–8. doi: 10.1016/j.hrcr.2025.10.005 (PMC12805267; doi:10.1016/j.hrcr.2025.10.005)
Supplement: Supplemental material [file mmc3.docx]

**Supplemental Video 1.** Propagation map of AT1. The activation pattern suggests a macroreentrant circuit around the left pulmonary veins. The posterior roof line appears blocked endocardially, with propagation likely occurring via an epicardial septopulmonary bundle. AT – atrial tachycardia; LA – left atrium.

**Supplemental Video 2.** Termination of AT2 into sinus rhythm after a few seconds of radiofrequency ablation at 20 W from the pulmonary artery. Power was subsequently titrated to 25 W and energy delivery was continued for 30 seconds to ensure lesion consolidation. AT – atrial tachycardia; RF – radiofrequency; PA – pulmonary artery.
